# Review of technologies for biomethane production and assessment of Eu transport share in 2030

**DOI:** 10.1016/j.jclepro.2019.02.271

**Published:** 2019-06-10

**Authors:** M. Prussi, M. Padella, M. Conton, E.D. Postma, L. Lonza

**Affiliations:** a European Commission, Joint Research Centre (JRC), Ispra, Italy; b Natural & Bio Gas Vehicle Association (NGVA Europe), av. de Cortenbergh 172, 1000, Brussels, Belgium

**Keywords:** Biogas, Biomethane, EU potential, Bio-LNG, Bio-CNG

## Abstract

The upgrade to biomethane allows extending biogas applications to transport sectors, supporting EU goals toward carbon neutrality. Biomethane produced from biogas upgrading can today rely on a large number of plants, estimated by the European Biogas Association in over 17000 in 2016, for a total installed capacity of 9985 GW (EBA, 2017).

After 2020, biogas and biomethane will count towards the 32% target of renewable energy share of the EU energy consumption, and towards a sub-target of minimum 14% of the energy consumed in the transport sector by 2030 (REDII).

In this framework, the paper aims to define the current market penetration of biogas upgrading technologies. A database has been created for EU-28, to highlight the relative importance of each technology. Based on the database, a EU production potential is defined, along with the EU demand potential for transport, in order to verify the match between supply and demand. The analysis of the current state of play of the sector suggests that a large potential can be foreseen for near future; the expected increase in biomethane production is based also on the possibility to convert residual feedstocks, such as Municipal Solid Wastes.

In this work a moderate technology penetration scenario has been set for EU-28, reaching a potential for biomethane of 18 billion m^3^/year in 2030. A large share of this potential can be devoted to transport, with a relevant impact on the sector. LNG appears suitable for pushing the market uptake of biomethane in the transport sector. Among the potential uses of biomethane for transport, it is worth considering that the natural gas use in the maritime and internal waterways sectors is getting momentum.

The current analysis aimed to highlight the potential of renewable energy-based alternatives to natural gas. Eventually, it is worth noticing that the real market deployment of this potential will be determined by the energy market conditions, and by the member states capability to stimulate the industry through a coherent set of supporting initiatives.

## Highlights

• A database of biogas upgrading plants is created, for EU-28.
• EU production potential is defined along with the EU demand for 2030 transport.
• A large potential can be foreseen for near future, based on residues and wastes.
• In a moderate scenario for EU 2030, biomethane production values 18 bcm/yr.

## List of abbreviation

AD: Anaerobic Digestion
bcm: Billion m^3^
CAPEX: Capital Expenditure (investment cost)
CIC: Italian certificates used for biomethane incentive (*Certificati di Immissione in Consumo)*
CNG: Compressed Natural Gas
CRY: Cryogenic separation
CSC: Chemical Scrubbing
DEA: Diethanolamine
EBA: European Biogas Association
GHG: Green House Gasses
HHV: Higher Heating Value
LCV: Light Commercial Vehicle
LHV: Lower Heating Value
LNG: Liquefied Natural Gas
FIP: Premium Feed-in tariffs
FIT: Feed-In Tariffs
MDEA: Methyl diethanolamine
MEA: Monoethanolamine
MEB: Membrane separation
MIX: More than one technology used for biomethane separation
MN: Methane Number
MS: Member State
MSW: Municipal Solid Waste
NI: Information not available
OPEX: Operating Expense
ORC: Organic Ranking Cycle
PCV: Passengers Car Vehicle
PSA: Pressure Swing Adsorption
PtG: Power to Gas
PZ: Piperazine
RED II: Renewable Energy Directive Recast
RG: Renewable Gas: the term encompasses biomethane, power-to-gas from renewable el. and SNG from biomass gasification
SNG: Synthetic Natural Gas
TSA: Temperature Swing Adsorption
VS/TS: Volatile Solids/Total Solids
WSC: Water Scrubbing

## 1 Introduction

The interest in upgrading biogas to biomethane has been growing in recent years, for its application in the electricity, heat as well as road transport sectors. According to the IEA outlook (IEA WEO, 2017), demand for natural gas is expected to grow in 2040 by more than half of the current consumption: the fastest rate among fossil fuels. Creating a carbon-neutral transport sector and establishing supply diversification are other two relevant targets for all countries (Pellegrino et al., 2017). Diversification in energy supplies is one pillar of the EC energy security strategy (EESS, 2014), which highlights the strong EU current dependence on foreign importers and, consequently, requests the Member States to develop diversification measures (Praks et al., 2015).

In the renewable energy and transport sector, biogas and biomethane represent effective strategies to move towards the targets set by the Renewable Energy Directive (2009/28/EC, RED) as amended by the ILUC Directive (Directive 2015/1513) for 2020, as well as to the targets for 2030 set in the compromise recently achieved on the recast of the directive (REDII). Biomethane currently counts towards the target of 20% renewable share of the final energy consumption from renewable sources by 2020, and to the target of 10% share of energy from renewable resources in each member state’s energy consumption in the transport sector. Until 2020, the use of biomethane in transport also can contribute to satisfy the goal to reduce the average GHG emissions from the production and use of fuels by 6% compared to a 2010 baseline as set in the Fuel Quality Directive (2009/30/EC, FQD). After 2020, biogas and biomethane will count towards the 32% renewable energy share from EU energy consumption and towards a sub-target of minimum 14% of the energy consumed in the transport sector by 2030 (2018/844/EC, REDII). Within the transport target, 3.5% must come from advanced biofuels produced from feedstocks listed in Part A of Annex IX that includes manure and sewage sludge, biowaste from households and industry, agriculture and forestry residues, algae, and energy crops among others. Advanced biofuels will be double-counted towards both the 3.5% target and towards the 14% target. Sustainability criteria for biofuels used in transport are defined by the directive - as well as for solid and gaseous biomass fuels used for power, heating and cooling sectors - and they must be fulfilled in order for the biofuels and bioenergy to account towards the above-mentioned targets.

In Europe, the number of biogas plants has been estimated in over 17 000 in 2016 (EBA, 2017). The European Biogas Association reported a total of 17 662 plants, of which 10 849 in Germany, 1 555 in Italy and 873 in France, for a total installed capacity of 9 985 GW. It is worth noting that, unlike other renewable energy plants (i.e. solar or wind), biogas installations have reached high availability (capacity factor), that allow for relevant energy production in kWh/yr per installed kW. The biomethane production is influenced by the biogas composition, which depends on the feedstock and the process used for its production; the methane content ranges from 45 to 60% in the case of landfills gas up to 60–70% for organic waste digesters (Ullah Khan et al., 2017). Biomethane produced by biogas upgrading needs to meet the gas quality specifications set by the European standard EN 16723-1 for injection into the gas grid or the quality specifications set in the standard EN 16723-2, to be used as BioCNG or BioLNG in road transport. Various estimations of biomethane potential have been carried out in recent years (i.e. van Grinsven et al., 2017); according to EBA (EBA, 2017), in 2016 Europe biomethane plants were 513, with an estimated production of 17264 GWh.

At national level, many countries use dedicated support mechanisms for the development of the biogas sector, including feed-in tariffs, premium feed-in tariffs and fiscal incentives. Feed-in tariffs are the minimum prices guaranteed, over a defined period, by the national governments for each kWh generated, either injected into a network or directly used. This incentive applies currently to Italy, Austria, France and United Kingdom. Another option is the Premium tariffs, which sets a premium on the existing electric power price; the producers obtain a revenue from the sale of energy in the electrical market and an additional one related to the premium tariff (Del Río et al., 2017). This is a technology-specific subsidy level per unit of renewable energy at a pre-set, fixed or floating rate. Tax exemptions or reductions are usually additional (and minor) support systems. As relevant case study, Italy issued on March 2018 the `Biomethane Decree’ (IT-MS, 2018), with specific targets for transport sector: if the biomethane is destined for the transport the incentive is a Certificate with an average value of 375 €; additional premiums are foreseen in case of installation of compression, liquefaction and/or distribution plants.

In this framework, the paper aims to review the current available technologies for biomethane upgrade, and define their current market penetration in the EU. This step required to develop a specific database, able to go beyond the current available literature. JRC collected data from various sources, structuring the dataset to highlight the share of each upgrading technology, for each plant size segment, etc. The database has been used to draw a 2030 scenario, for estimating the EU production potential. This potential has been eventually compared the outcomes of other studies setting the expected natural gas demand for transport sector, with the aim to estimate the potential impact of biomethane.

### 1.1 Review of the current available technologies for biogas upgrade

Upgrading biogas to biomethane can be performed by means of various technologies, largely derived from other sectors (e.g. cryogenic separation of gases for medical or other industrial applications). These technologies include physical and chemical absorption, adsorption, membrane and cryogenic separation. Other biological pathways could be considered suitable for biogas upgrading but their level of maturity is currently lower than the above-mentioned technologies. The technical availability, defined as the yearly percentage of the operative hours, is a fundamental parameter for the separation step: an upgrading technology is being considered ready for the market, when its capacity and reliability can be compared with the one of the existing biogas plant. Upgrading technologies available today at commercial scale can be listed as: Pressure Swing Adsorption, Water scrubbing, Chemical scrubbing, Membrane separation, Cryogenic separation.

#### 1.1.1 Pressure Swing Adsorption

PSA is a technique based on the selective adhesion of one or more components of a gaseous mixture, on the surface of a micro-porous solid; the material for biogas upgrading is typically equilibrium-base adsorbents. The pores of the adsorbent should allow an easy penetration of the CO_2_ molecules, whereas filtering the larger CH_4_ molecules. Molecular sieve materials such as zeolites and activated carbon are commonly used as adsorptive materials for biogas upgrading (Zhao and Leonhardt, 2010). There are several companies, active on EU market, able to supply this technology, for low and high capacity (biogas flowrate from 10 to 10000 m^3^/h). Taking into consideration that the materials used in PSA plants are sensitive to fouling, by the impurities in the biogas stream (Augelletti et al., 2017), a pre-treatment step is typically required. This upgrading technology allows achieving high methane concentrations: 95–99%, thus being able to comply with the typical technical specifications for the grid injection. The necessity of pre-treatment is a drawback of this technology, together with the required extensive process control and the high costs. In order to reduce the operational costs, an interesting solution is the use of the temperature swing adsorption (TSA). TSA works at constant pressure, and it requires thermal energy to regenerate the adsorbent material, being suitable for applications where cheap source of heat is available (Khan et al., 2017).

#### 1.1.2 Absorption techniques

Absorption techniques are based on the solubility of the gases contained in biogas, in a certain liquid. Typically, either water or organic solvent (e.g. methanol, N-methyl pyrrolidone, and polyethylene glycol ethers) are used to absorb CO in physical absorption plants, whereas amine scrubbing is widely used for chemical absorption (Rotunno et al., 2017). Water scrubbing is used as an upgrading technique as well as a pre-treatment (e.g. before PSA) for the removal of H_2_S. The main limit of the WSC is that significant plant size is required to achieve high final concentration of methane.

Chemical scrubbing (CSC) involves reversible reactions between absorbed substances and solvent. The most common solution for biogas upgrading is based on amines: diethanolamine, monoethanolamine, methyl diethanolamine and piperazine (Chen et al., 2015). Amine scrubber consists of an absorber tank, where the CO_2_ is absorbed from the biogas (operating at 20–65 °C and 1–2 bar), followed by a stripper in which the CO_2_ is released by heating the stream. CSC with amine allows to reach a highly concentrated biomethane stream CH_4_>99%. CSC requires a pre-treatment stage, in order to remove H_2_S. CSC is today characterized by high operational and investment costs (Angelidaki et al., 2018).

#### 1.1.3 Membrane separation

Membrane are permeable barriers, specifically designed to be selective to specific molecules; process drivers are relative concentration, pressure, temperature, and electric charges of the different molecules. On the market, three types of membranes are typically used: polymeric, inorganic, and mixed matrix membranes. Inorganic membranes have several advantages compared to polymeric, mainly due to their higher mechanical strength, chemical resistance and thermal stability (Chen et al., 2015). The current trend in industrial applications is to use mixed matrix membranes (Khan et al., 2017). As for other technologies, pre-treatment is needed in the case of MEB plants, as H_2_S negatively affect medium –term performance. A multistage membrane strategy is typically adopted to recover CH_4_ up to 99.5% (Chen et al., 2015). Costs and reliability are the major factors today limiting MEB market penetration.

#### 1.1.4 Cryogenic separation

Cryogenic separation is a well establish technology for gas separation, for various large-scale industrial applications. The physical principle behind cryogenic technique is that gases like CO_2_ and H_2_S liquefy under different pressure and temperature conditions: cryogenic plants operate under a very low temperature (−170 °C) and high pressure (80 bar). Biogas purification can be performed by cryogenic technology, obtaining the lower methane losses but current scale-down factors are increasing the specific costs (Sahota et al., 2018). Despite the higher costs, cryogenic process is still of interest for producing road fuel, as it allows to produce liquefied natural gas (Bio-LNG).

#### 1.1.5 Biological upgrade techniques

An interesting alternative to the current biogas upgrading techniques is represented by biological methods (Lee et al., 2012), (Kougias et al., 2017), namely biological separation via hydrogenotrophic methanogenesis and consisting in the use of hydrogenotrophic-methanogens to convert CO_2_ and H_2_ in CH_4_. Despite the potential advantages of these techniques, challenges are today limiting the market deployment and the current practical interest for the sector.

## 2 Material and methods

JRC-Ispra has developed a database of the most relevant biomethane initiatives around Europe. The database has been created by collecting and structuring information from various sources, in particular from projects websites, dataset provided by associations, literature, etc. (Hoyer et al., 2016), (Angelidaki et al., 2018), (Deremince, 2017), (GIE and EBA, 2018), (RBN, 2018) and (DENA, 2018). These various sources contained both aggregated as well as disaggregated data, with non-homogenous information about plant size, feedstock input, technologies used for biogas upgrading, etc. The creation of an organic set of information has required to cross-check the various sources, and standardize the information.

A basic criteria for selecting the plants for the database has been the size of the initiative. As database aims to draw a picture of the current and future European biomethane industrial potential, small pilot initiatives have not been considered; the threshold has been a nominal biomethane production capacity higher than 10 m^3^/h.

In the database, each plant is described by the following characteristics:

• plant location,
• feedstock used for biogas production,
• the nominal productivity,
• the technology for biomethane separation.

Nominal productivity refers to the maximum flow rate of biomethane declared by the owner, on the base of the technical feature of the separation stage (or by the limiting dimension of any other plant device). When the available information about productivity resulted unclear, the lower production figure has been chosen to populate the database. Technologies used for biomethane separation have been described in the previous section, and they constitute a fundamental information for the database: a mandatory input for each plant. The data are organized in a pivot structure in Excel, in order to be able to extract dataset segmented per technology, country, feedstock type, etc.

Some aggregated values - such as the total production for specific countries - available from other sources, than those used for the database creation (i.e. (IEA T37, 2016)), have been utilized for validation.

## 3 Results and discussion

### 3.1 Survey of existing EU plants for biomethane production in EU

**Fig. 1:**
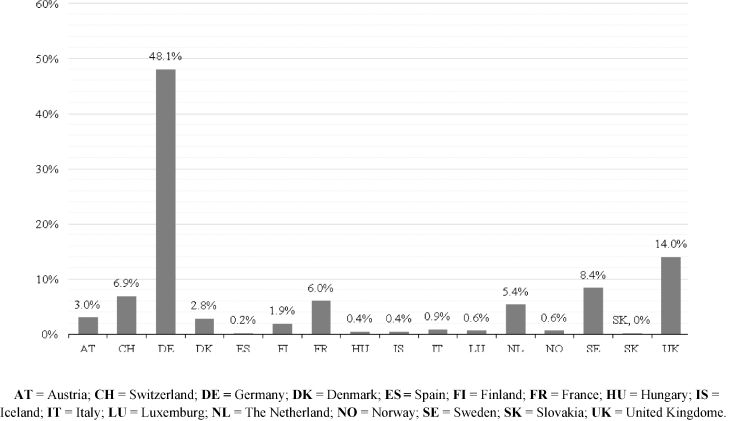
Country segmentation on total EU installed capacity.

According to the JRC database (basic info available in annex I), the total number of operative relevant plants in EU-28 is 465. This value differs from other sources, such as EBA (EBA, 2017) possibly for the different criteria used to identify commercial plants with respect to the pilot/research initiatives. Fig. 1 shows the number of plants recorded in the JRC database, segmented per country. Germany is the country currently leading the sector, with more than 200 plants spread on its territory. UK, France and Sweden are also active in the field; surprisingly, despite the large number of biogas plants, Italy does not have a significant number of upgrading plants already in operation.

**Fig. 2:**
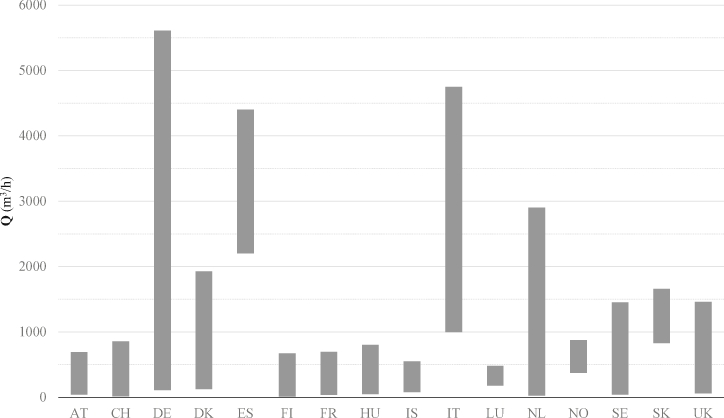
Maximum and minimum biomethane production capacity per country.

**Fig. 3:**
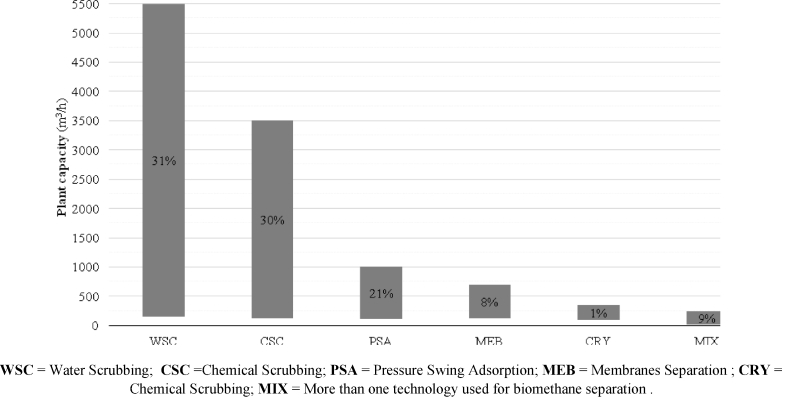
Typical range (max and min plant capacity) for each upgrading technology, and relative share on the total current EU installed plants.

Based on the nominal installed capacity, Fig. 2 shows the maximum and minimum potential flow of the operative plants in each country. The size of the plant has a direct correlation to the technology used for biogas upgrading and to the level of market penetration in the specific country: very small plants suggest the presence of pilot activities. Fig. 3 reports the share of each technology as a percentage of the total EU-28 plants. It emerges that chemical scrubbing, water scrubbing and pressure swing absorption represent today more than 2/3 of the market. Market penetration of each technology is related to their capability of being scaled down compared to other commercial applications (e.g. production of liquid gases) to the typical size of biogas plants. Not surprisingly, WSC is the most flexible application, while the CRY suffers for the high CAPEX when applied to small-scale plants.

### 3.2 Future EU biomethane technical production potential

Today there are several potential pathways for producing alternatives to natural gas; namely biomethane from biogas upgrade, natural gas by power-to-gas from renewable electricity and CO_2_ and Synthetic Natural Gas from biomass gasification. Power-to-gas is a pathway designed to use the peaks of renewable electricity to produce Hydrogen and using it for fixing CO_2_ via methanation reaction (Götz et al., 2016); whereas, SNG refers to biomass gasification and syngas upgrading to synthetic natural gas by thermochemical processes (Duret et al., 2005).

JRC has recently investigated the deployment status of technologies. Notably, for SNG from biomass gasification, the cancelling of the EU largest initiative – the GoBiGas project (BioEnergyInternational, 2018) - clearly highlighted the difficulties that this technology has been facing, though the on-going remarkable exception constituted by the AMBIGO initiative (Overwijk et al., 2018). Interestingly, a shift in stakeholder attention has been observed, as the scientific and industrial communities seem currently focusing on methane synthesis not via biomass route but as a promising technology for the power-to-fuel applications.

Focusing on biomethane production from biogas upgrade, based on the data available in JRC database, the nominal capacity currently installed in EU-28 accounts for 236 000 m^3^/h. In order to calculate the annual production potential, the biogas plant availability (in terms of operational h/yr) is a key parameter: it has been proven to be very high and upgrading plants is showing technical availability up to the 96% (Bauer et al., 2013) and thus the annual potential energy output can be calculated considering 8410 h/yr. The resulting annual nominal potential for biomethane can be estimated in 1.98 billion m^3^/yr, equivalent to 71.7 PJ (calculate on HHV). Based on the calculated potential, a moderate scenario has been defined for the market penetration uptake of biomethane in 2030. As presented in Table 1, biomethane is expected to grow rapidly, as the technologies have already been demonstrated at significant scale and the infrastructure for the product substantially developed. Biogas plants in many countries (i.e. Italy) are expected to shift from energy generation to biomethane production, once the previous incentive support will come a natural conclusion. In the scenario proposed, the overall biomethane potential for EU at 2030 accounts for about 18 bcm/yr.

**Table 1:** Moderate scenario for biomethane.

[bcm/yr]	2017	2020	2025	2030
Biomethane	1.9	9.0	15.5	18.0

This estimation results more conservative than those from other studies; for instance, TU-Delft reported (CE DELFT, 2016) a 2030 production potential of biogas from waste and residues streams ranging from 33.6 to 46.9 billion m³/yr, representing 2–4% of the estimated total primary EU energy consumption. The results’ variability depends on the amount of feedstock deployed and the assumed learning effect curve.

A recent report from NGVA (NGVA-a, 2018) estimates an even larger potential at 2030, in the range of 36–51 billion m³/yr (Table 2). The study looks upon availability of sustainable feedstock and power-to-fuel medium term deployment as important parameters; this considers a larger use of animal manure: the current biogas production from animal manure is reported to be 3.3% of the total EU potential. In the same study, a large biogas potential is expected to derive from garden waste and biowaste from households and agro-food industrial processes thanks to the higher biowaste recycling targets set in the recently revised Waste Framework Directive (EU, 2018/851). Following the sector trends, NGVA took into consideration a wide use of straw: equal to 40% of the total EU straw potential (remaining 60% for other uses including leaving on field) (Antonczyk et al., 2016), which would provide 3.7 billion m^3^of raw biogas (Sternberg et al., 2017). Moreover, the NGVA report considers a significant contribution from technologies which are alternative to fermentation, such as Power-to-gas and Synthetic Natural Gas from biomass gasification.

**Table 2:** Production forecasts for renewable gas by NGVA (NGVA-b, 2018).

[bcm/yr]	2017	2030
Anaerobic Digestion	1.8	13–19
Power-to-Gas	8.0 10^−3^	11–16
SNG	3.0 10^−6^	12–16
**TOTAL**	**1.8**	**36**–**51**

### 3.3 Current and potential biomethane transport sector demand in the EU 2030

Biomethane is considered as a suitable alternative to fossil natural gas for two main applications: direct injection into natural gas grid and use in transport. Countries such as Italy have a well spread gas infrastructure: already in 2014, more than 32 000 km of grid (SNAM, 2014) and numerous connections to other transnational grids were available. This asset can support biomethane penetration, but meeting quality standards remains a fundamental objective for the sector (Svensson, 2014). Several countries have already defined their own standards, such as Italy (UNI/TR 11537:2016), Sweden, Germany, Switzerland and France. Thanks to the effort put in the harmonization process, there are two standards currently valid at EU level: EN 16723 – part I and part II. The first part describes the definition of the standard for grid injection, whereas the latter refers to automotive use.

**Fig. 4:**
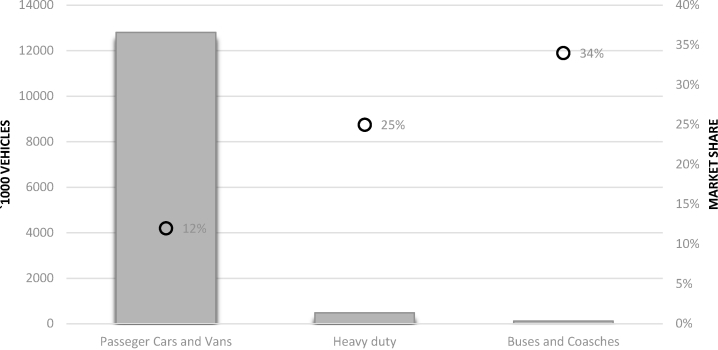
Elaboration from NGVA’s gas vehicle roadmap towards 2030 (NGVA Europe, 2018).

If quality standards are meet, transport sector is a promising option for valorising biomethane; NGVA (CNG-EU, 2018) estimates the current number of CNG vehicles in EU-28 in about 1.3 million light duty vehicles, 17 000 buses and coaches and 9000 trucks. In the same study, the number of methane fuelled trucks in 2030 are set in 480 000 units, thus with an expected average year on year new sales of about 36 000 units (Fig. 4).

Besides CNG, LNG appears a suitable option for pushing the market uptake of biomethane in transport as, by increasing the biomethane energy density, it can significantly extend the operational range of trucks; several big sector players are rapidly moving towards commercialization of LNG trucks (IVECO, 2018). Moreover, in several non-EU market, such as Asia, LNG is getting attentions and many initiatives are on-going (Ecofyn, 2016). On the production side, among the various separation technologies, cryogenic is particularly promising as it allows the direct production of LNG but installation costs are limiting their market deployment. The expected market penetration of CNG and LNG supplied vehicles account for the 12% for PCVs and LCVs, 34% for buses and coaches and 25% for trucks, in 2030, corresponding to a total consumption of about 30 billion m^3^/yr. As biomethane is expected to be used also for other applications than transport, NGVA set a scenario in with the 30% of their expected biomethane production in 2030 (36-51 bcm/yr, see Table 1) to road sector, which 3 account for about 9 bcm/yr (NGVA-b, 2018).

This estimation are confirmed by other studies, such as the Roland Berger (Roland Berger, 2016), which foresees an increasing share of alternative powertrains in commercial vehicle segment; in particular: LNG will be the most important alternative powertrain in the heavy duty segment by 2030 with a share of about 10% in new registrations; CNG will contribute to medium duty vehicle segment (+4% of the new sales in 2030) and will results fundamental for bus segment (14% of new registration in 2030).

Based on the work carried out, the demand drawn for transport sector appears to be larger than the expected production potential (18 bcm/yr), but still biomethane could represent an important contribution.

Additionally, the use of biomethane in transport sector is not necessarily limited to road segment; it is worth considering that the natural gas use in the maritime and internal waterways sectors is rapidly getting momentum. JRC carried out an exploratory study aiming to try to set a value for the potential demand for marine and inland waterway alternative fuel (EU marine, 2016); the outcome of the study described a current stationary situation but with a potential for LNG in the medium term.

In order to define a future potential deployment for the CNG vehicle sector, infrastructure represents a key step. The European Commission, in the framework of the Directive on the deployment of alternative fuels infrastructure (EC, 2014/94/EU) requires that Member States provide a minimum infrastructure for alternative fuels such as electricity, hydrogen and natural gas. EAFO (EAFO, 2018) reports 3351 CNG refuelling stations in 2016. NGVA estimates 3426, in 2018, with Italy leading the scene with more than 33% of the share; LNG filling stations are also growing rapidly, reaching in 2018 155 operating points (NGVA-b, 2018). The real market penetration of CNG and LNG will also be affected by Member States capability to create the proper infrastructures, as reported in the National Policy Framework working document (NPF, 2017); there are MS setting ambitious targets, while others express pessimistic view about higher CNG penetration.

## 4 Conclusions

The analysis of the current state of play of the biogas upgrading sector suggests a current production potential of 1.9 billion m^3^/yr. The total number of commercial initiative in Europe is about 645, in 2017; with Germany leading the scene. Upgrading technologies are mainly based on three techniques: PSA, WSC and CSC. New biogas plants will likely be equipped with biogas separation units, which allow fostering costs reduction and a parallel consolidation of the operation plants availability. A larger production can be expected in the near future: 18 billion m^3^/yr in 2030; mainly based on the improvement of the already installed biogas plants, possibly fed with residual feedstocks: for instance MSW. It is worth noticing these potential accounts for around 10% of EU’s natural gas import projected for 2030 (EC, EU Reference Scenario, 2016).

The current analysis aimed to highlight the potential of biomethane production, as renewable energy-based alternative to natural gas for transport sector. Road and marine transport sectors are likely to absorb a large share of this EU biomethane production: a demand of 30 bcm/yr of natural gas in 2030 is expected by road mode (NGVA-b, 2108), (Roland Berger, 2016).

Eventually, it is worth noticing that the real market deployment of EU biomethane production potential will be determined both by the energy market conditions, but also by the member states capability to create infrastructure, and stimulate industry by a coherent set of supporting initiatives.

## Disclaimer

The views expressed here are purely those of the authors and may not, under any circumstances, be regarded as an official position of the European Commission.
